# Demographic Patterns, Risk Factors, and Outcomes of Abdominal Aortic Aneurysms in Young Adults ≤55 Years: An Experience in a Tertiary Care Centre of India

**DOI:** 10.7759/cureus.17372

**Published:** 2021-08-22

**Authors:** Arunanshu Behera, Cherring Tandup, Swapnesh K Sahu, Lileswar Kaman, Ajay Savlania, Anil L Naik, Shibojit Talukder, Basant Singh, Bramhadatta Pattnaik, Krishna Ramavath

**Affiliations:** 1 General Surgery, Post Graduate Institute of Medical Education & Research (PGIMER), Chandigarh, IND; 2 Hepato-Pancreatico Biliary (HPB) Surgery, Addenbrookes Hospital, Cambridge University Hospitals NHS Foundation Trust, Cambridge, GBR; 3 Surgical Gastroenterology, All India Institute of Medical Sciences, Patna, IND; 4 Surgical Gastroenterology, All India Institute of Medical Sciences, Bhubaneshwar, IND

**Keywords:** aortic aneurysm, abdominal, young adult, demographics, risk factor, outcome study

## Abstract

Introduction

Abdominal aortic aneurysms (AAA) are uncommon in young adults ≤55 years of age. There is a lack of literature on clinical characteristics, risk factors, and therapeutic outcomes so we present a case series of 11 patients of AAA aged ≤55 years.

Methods

We included single-center retrospective case series between 2013 to 2020. We reviewed 44 patients who were operated for AAA in a tertiary care center in India. We identified 13 patients who were ≤55 years; two patients with incomplete records were excluded. A patient information sheet was used to retrieve demographic data, clinical presentation, outcomes, and follow-up.

Results

Out of 11 patients, 10 were men. Nine patients (81.8%) had symptomatic AAA. The majority (45.4%) exhibited an infrarenal aneurysm and the median size of the aneurysm was 5.8 cm (IQR: 5.5-6.4 cm). Eight patients (72.7%) had a history of smoking. Hypertension was observed in six patients and one patient had associated coronary artery disease. Clamping time was > 45 minutes among three patients; all smokers. Blood loss was > 500 ml in five patients. The median length of hospital stay was 10 days (7-40); more among patients with metabolic equivalents (METS) score < 4, 14.5 (8-19) days. No grade III-IV complications and mortality were noted with a median follow-up of 15 months, with all patients living.

Conclusion

The aneurysm was symptomatic in the majority of participants. An association of smoking in increasing both the median clamping time and length of hospital stay was seen. No mortality and good disease-free follow-up suggested good outcomes.

## Introduction

Abdominal aortic aneurysm (AAA) is a pathological permanent dilatation of an aorta, more than 1.5 to 2 times its original size of approximately 2 cms, in the abdominal area [1]. It is localized infra-renally in 88%-89% of individuals [2]. General demographics are available for developed countries, such as Northern Europe, the United Kingdom, and Australia, where their estimated prevalence is greater than 5% [2-3]. It is estimated that approximately 0.6 million individuals in India were affected by AAA in 2016, with a prevalence of 0.4% [4]. These findings suggest that the prevalence of AAA is lower in Asian countries or significantly underreported.

Increasing age, male predilection, physical inactivity, smoking, cardiovascular disease, high blood pressure, and family history are significantly associated with abdominal aortic aneurysms [3,5-6]. Cocaine, amphetamines, dyslipidemia, and certain genetic diseases also predispose to the formation of aneurysms [7]. AAA is asymptomatic in young patients compared to older patients and is generally in the form of ischemia of the limbs, abdominal pain (in case of rupture), or pulsating abdominal mass (not ruptured) [8]. Asymptomatic cases are usually incidentally diagnosed during abdominal imaging procedures like X-rays, CT, or MR.

In older people, rupture of AAA leads to a very high mortality rate, ranging from 65% to 85%, with a cause-specific mortality rate of 1.3% [6]. The risk of rupture is rare in young adults but the chance of mortality remains high with delayed diagnosis and treatment. Available treatment modalities include open aortic repair (OAR) using a prosthetic graft and endovascular aortic repair (EVAR) using a grafted stent, each with its own benefits and limitations [9]. The morbidity and mortality data are usually for the elderly, as the presence of AAA in people under the age of 55 is rare. Though there have been reports of scattered cases of AAA among younger adults, studies do not present any conclusive findings on the presenting symptoms, risk factors, surgical and management outcomes, along with documented complications [10].

We have tried to delineate the etiological factors, presenting symptoms, and outcomes of patients aged ≤55 years, who underwent open repair AAA surgery in a tertiary care hospital of North India. We studied this age group, as various literature is available for the age group of more than 55 years and no large series of young adults presenting with AAA is present.

## Materials and methods

We reviewed previous medical records for patients who underwent AAA surgery in the past seven years (2013 to 2020) at our center. Patients under 56 years of age were included. This study was authorized by the institutional ethics review committee and registered under IEC No. INT/IEC/2021/SPL.864 dated May 25, 2021. The manuscript is in accordance with the Helsinki Declaration and with local ethical guidelines; informed consent was taken where applicable. Demographics related to age, sex, and risk factors, including smoking and related diseases (hypertension, cardiovascular disease, diabetes mellitus, pulmonary arterial disease, chronic obstructive pulmonary disease (COPD), chronic kidney disease), were recorded. The presence of AAA was determined from ultrasound imaging and contrast-enhanced computed tomography, the extent of the aneurysm was assessed through preoperative computed tomography and confirmed intraoperatively. Follow-up information was provided, and we followed the Society of Vascular Surgery guidelines for the care of patients with abdominal aortic aneurysms [11].

All patients received open repair treatment using a transperitoneal approach under general anesthesia. After exposure to AAA, proximal and distal clamping was carried out and the aneurysm was dissected. Either Dacron or polytetrafluoroethylene (PTFE) graft was used to construct the neo-abdominal aorta as per the surgeon’s preference. The intraoperative blood loss, clamping time of the proximal aorta, and the graft material used were recorded as mentioned in the institution's records. Perioperative mortality and morbidity data were available for each case and recorded as per the Clavien-Dindo classification [12]. Data were entered and analyzed using SPSS v24 (IBM Corp., Armonk, NY). Descriptive analysis was performed for social-demographic distribution, comorbidities, risk factors, clinical profile, interpretive considerations, treatment complications, and outcomes during follow-up. The duration of stay in hospital and the duration of follow-up were studied according to gender, the presence of comorbidities, and the location of the aneurysm. The distribution of comorbidity in the presence and absence of smoking has also been investigated.

## Results

**Figure 1:**
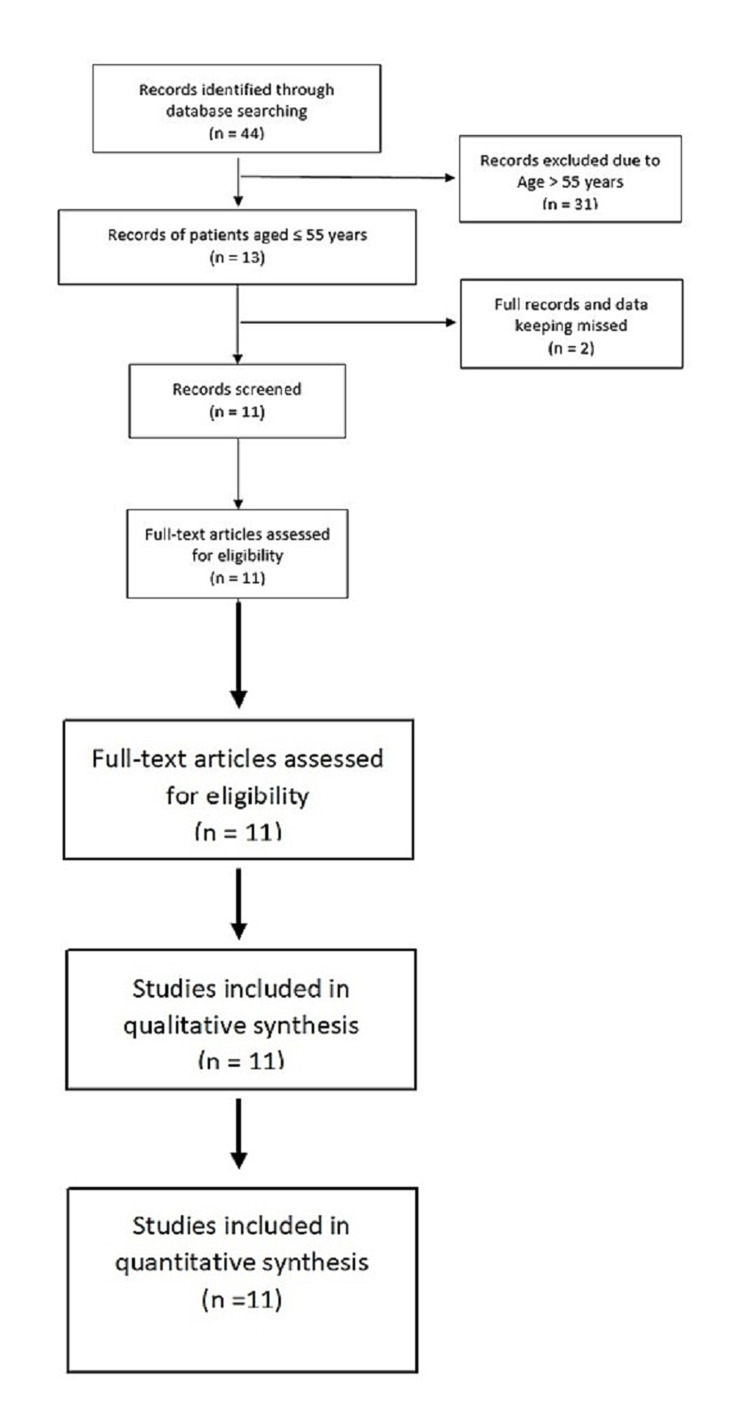
Flowchart showing the patients enrolled retrospectively

**Figure 2:**
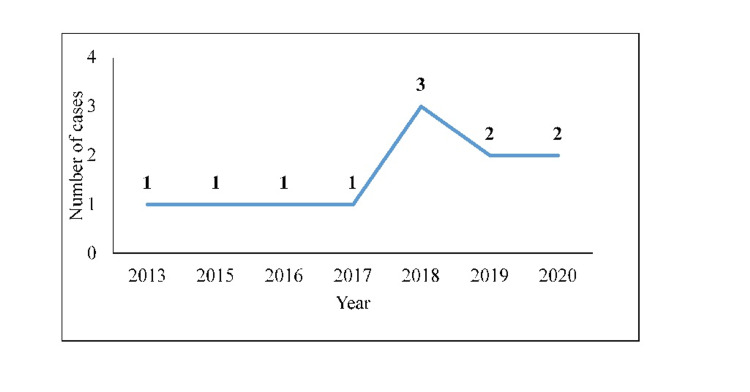
Time trend of abdominal aortic aneurysm over eight years (2013-2020)

**Figure 3:**
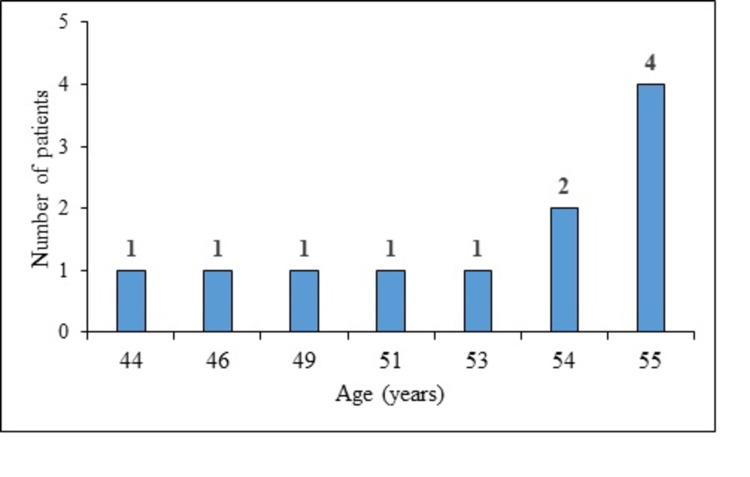
Age-wise distribution of the participants

From 2013 to 2020, data of 44 patients who underwent surgery for an abdominal aortic aneurysm were reviewed. We identified 13 patients who were ≤55 years, two patients had incomplete records, and were excluded so a total of 11 patients were included (10 males, 1 female) under the age of 56 years underwent AAA surgery via open repair (Figure 1). The time trend indicated a low disease incidence, ranging from one to three every year, with no case in 2014 (Figure 2). The median age of patients was 54 (49-55) years. The number of cases increased with the age progression (Figure 3).

**Figure 4:**
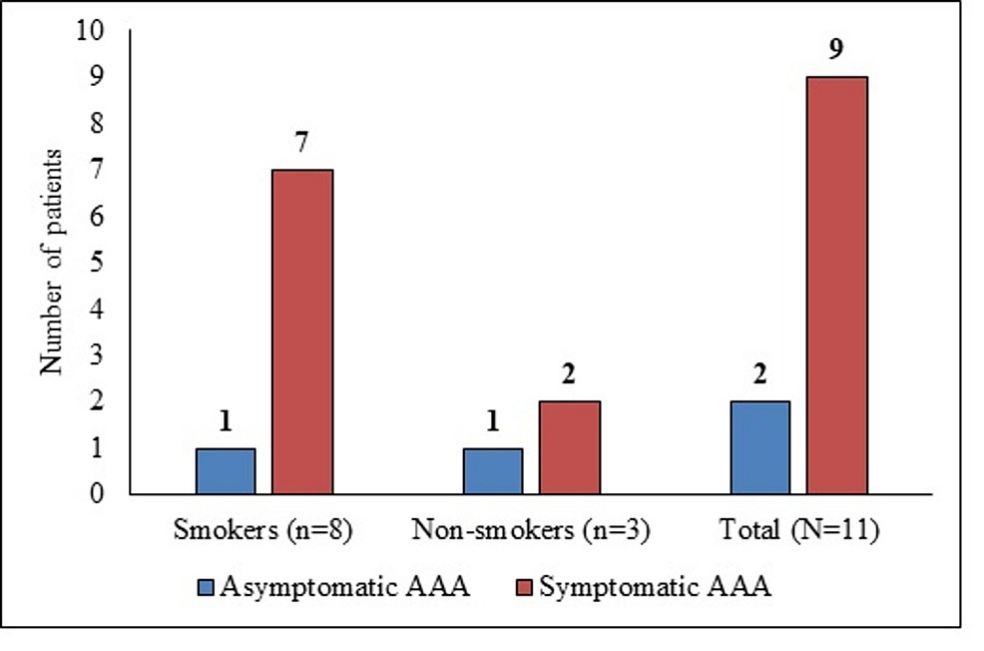
Distribution of symptomatic and asymptomatic AAA among smokers and non-smokers AAA- abdominal aortic aneurysm

Seven (63.6%) patients had a metabolic equivalent (MET) score of more than four indicative of moderate to vigorous physical activity. Eight (72.7%) patients were smokers (Figure 4). Six patients (45.5%) were known hypertensive of which one patient had dual comorbidity of hypertension and coronary artery disease (CAD). None of the patients suffered from pulmonary artery disease, diabetes mellitus, chronic obstructive pulmonary disease, or chronic kidney disease. Overall, six (54.5%) patients presented with comorbidities (Table 1). Among the smokers (n=8), five (62.5%) patients had hypertension. Among non-smokers, only one (33.3%) patient had the dual comorbidity of hypertension and CAD. Moderate to vigorous physical activity was more prevalent among smokers (n=6, 75%) (Table 2).

**Table 1:** Summary of patients presenting with AAA AAA- abdominal aortic aneurysm; METS- metabolic equivalents; M- male, F- female; HTN- hypertension, CAD- coronary artery disease; SR- suprarenal, JR- juxtarenal, IR- infrarenal; BF- bifurcated, T- tubular; N- no, Y- yes; PTFE- polytetrafluoroethylene; SSI- superficial skin infection, I- ileus; CD- Clavien-Dindo grading system of complications; LOH- length of hospital stay (in days)

Sr. No.	Age/ Sex	Year	Risk factors			AAA characteristics		Intraoperative considerations						Postoperative considerations				Follow-up		
								Graft			Aortic clamping									
			METS	Co-morbidity	Smoking	Location	Symptoms	Material	Size (mm)	Shape	Time (min)	Location	Blood loss (ml)	ICU stay	Complication	CD	LOH (day)	Duration (months)	Status	
1	46/M	2013	>4	None	No	JR	Yes	PTFE	20	BF	<45	IR	<500	N	SSI	Y	7	60	Alive	
2	49/M	2015	>4	HTN	Yes	IR	No	PTFE	20	BF	<45	IR	<500	N	I	Y	12	38	Alive	
3	54/M	2016	<4	CAD, HTN	No	JR	No	PTFE	22	T	<45	IR	<500	N	N	N	20	40	Alive	
4	54/M	2017	<4	None	Yes	IR	Yes	PTFE	20	T	<45	IR	>500	N	N	N	7	34	Alive	
5	51/M	2018*	>4	HTN	Yes	IR	Yes	PTFE	18	T	<45	IR	<500	N	N	N	8	15	Alive	
6	55/M	2018*	>4	HTN	Yes	IR	Yes	PTFE	18	T	>45	IR	>500	Y	N	N	7	16	Alive	
7	55/M	2018*	>4	None	Yes	IR	Yes	Dacron	20	BF	<45	IR	<500	N	N	N	10	15	Alive	
8	55/F	2019*	<4	None	No	JR	Yes	PTFE	22	BF	<45	SR	>500	N	I	Y	18	14	Alive	
9	44/M	2019*	>4	HTN	Yes	SR	Yes	Dacron	20	T	<45	IR	>500	Y	N	N	8	10	Alive	
10	53/M	2020*	>4	None	Yes	SR	Yes	PTFE	20	T	>45	SR	>500	Y	SSI	Y	40	6	Alive	
11	55/M	2020*	<4	HTN	Yes	JR	Yes	Dacron	20	T	>45	IR	<500	N	N	N	11	9	Alive	

**Table 2:** Physical activity score and comorbidity status among smokers vs. non-smokers

Physical activity score and comorbidity status		Smoking status				Total (N=11)	
		Smoker (n=8)		Non-smoker (n=3)			
		n	%	n	%	n	%
Comorbidity status	Absent	3	37.5	2	66.7	5	45.5
	Present	5	62.5	1	33.3	6	54.5
Physical activity level	Light to moderate	2	25.0	2	66.7	4	36.4
	Moderate to vigorous	6	75.0	1	33.3	7	63.6

Nine (81.8%) patients presented with mild to moderate pain in the abdomen (Figure 4). The other two were diagnosed incidentally (Table 1). One patient was diagnosed while we were operating on him for gall bladder, and the other patient was diagnosed while getting the MRI done for some other abdominal pathology. AAA was located infrarenal in five (45.4%), juxtarenal in four (36.6%), and suprarenal in two (18%) patients. The median largest diameter of the aneurysm was 5.8 cm (IQR: 5.5-6.4 cm) and the mean diameter was 5.97±0.75 cm (Table 3).

**Table 3:** Diameter of abdominal aortic aneurysm (AAA) The median size of AAA was 5.8 cm (IQR: 5.5-6.4 cm) or 58 mm (IQR: 55-64 mm) [mean: 5.97±0.75 cm or 59.7±7.56 mm].

Size of AAA	Median (IQR)	Mean±SD
In cm	5.8 (5.5-6.4)	5.97±0.75
In mm	58 (55-64)	59.7±7.56

A PTFE graft and Dacron graft were used for eight (72.7%) and three (27.3%) patients, respectively. We used a tubular graft in seven (63.6%) and a bifurcated graft in four (36.4%) patients. The median diameter of the graft was 20 mm (range: 18-22 mm). Among eight (72.7%) patients, clamping time was less than 45 minutes. In six (54.5%) patients, intraoperative blood loss was less than 500 ml (Table 1). In patients with less than 45 minutes aortic clamping duration (n=8), five (62.5%) were smokers, seven (87.5%) had infrarenal clamping, six (75%) received PTFE grafts, five (50%) each received a bifurcated and tubular graft. All patients with more than 45 minutes duration of aortic clamping were smokers (n=3), two (66.7%) of them had infrarenal clamping, two (66.7%) received a PTFE graft, two (66.7%) received a 20 mm diameter graft, and all received tubular grafts (Table 4). We compared the outcomes.

**Table 4:** Distribution of smoking status and intraoperative findings with respect to aortic clamping duration PTFE: polytetrafluoroethylene

Variable		Clamping duration				Total (N=11)	
		Less than 45 mins (n=8)		More than 45 mins (n=3)			
		n	%	n	%	n	%
Smoking status	Smoker	5	62.5	3	100.0	8	72.7
	Non-smoker	3	37.5	0	0.0	3	27.3
Clamp location	Infrarenal	7	87.5	2	66.7	9	81.8
	Suprarenal	1	12.5	1	33.3	2	18.2
Graft type	Dacron	2	25.0	1	33.3	3	27.3
	PTFE	6	75.0	2	66.7	8	72.7
Graft shape	Tubular	4	50.0	3	100.0	7	63.6
	Bifurcated	4	50.0	0	0.0	4	36.4

Postoperatively, two (18.2%) patients suffered from ileus, and two others (18.2%) developed a superficial skin infection. Complications were classified as per the Clavien-Dindo grading system of surgery-associated complications. Seven patients did not develop any complications (Table 1). The median length of hospital stay (LOHS) was 10 (7-40) days; 9 (7-40) days for males and 18 days for a single female. The LOHS was more among patients with a MET score less than four with a median (IQR) of 14.5 (8-19) days as compared to patients with MET score more than four having a median (IQR) of 8 (7-12) days. Independent t-test done showed no difference in median hospital stay between patients with and without co-morbidity and having different levels of physical activity. There was no difference in the duration of hospital stay according to the location of the aneurysm. The median LOHS was longest for suprarenal AAA (40 days), followed by juxtarenal (14.5; 8-19) days, and the shortest for infrarenal location 8 (7-11) days. The presence or absence of co-morbidity did not affect the LOHS. 

The median duration of follow-up was 15 (10-38) months. Patients with suprarenal AAA were followed up for a median duration of eight months, juxtarenal for 27 (10-55) months, and the follow-up duration for the infrarenal location was 16 (15-36) months. During follow-up, no mortality was reported in our patients (Table 1).

## Discussion

An AAA results from an inflammatory injury to the aortic wall due to multiple risk factors and is common among people aged 55 years and older. A large portion of the literature abounds in treatment outcomes and reported complications among the elderly population. The etiology of the disease in terms of risk factors and management outcomes remains a gray area of clinical research in young adults suffering from AAA. Through this case series, we have presented the clinical course, presentation history, along with treatment outcomes in terms of intra- and postoperative complications, and the mortality associated with AAA among younger adults. Kun Li and colleagues did screening for AAA in at-risk residents of middle China, where participants of age more than 40 years were included; the study showed a higher AAA prevalence in the 55-75 years age group [13].

We analyzed data of 11 patients who underwent surgery for AAA since 2013-2020, aging up to 55 years. We found that the incidence of AAA was 2.6 times higher among smokers (n=8) than non-smokers (n=3) and 1.2 times higher among people with comorbidities (n=6). The occurrence of AAA among persons less than 55 years of age is uncommon, however, smoker males develop aneurysms earlier than elderly non-smoker males [14]. An Indian study involving 40-80-year-old individuals reported an overall higher prevalence of AAA among males, hypertensive individuals, and smokers [15]. However, they did not present the results on the basis of age so differences in clinical presentation between young adults and the elderly could not be described in their study. Similar risk factors have also been reported in scattered case reports among young adults around the globe [16-17]. It has also been shown that the risk factors for acute lung injury (ALI) among young adults and seniors remain the same [8,18].

In this study, nine cases had pain in the abdomen and two rest cases were accidentally diagnosed. No patient has had a ruptured aneurysm, which is otherwise common in the elderly [8,19]. This is an important finding that markedly differentiates the clinical presentation of AAA in young adults from the elderly. The order of aortic segment involvement in AAA is reported to be infrarenal (75.6%) > renal (58.3%) > suprarenal (43.9%), similar to our findings, but with different proportions, i.e. infrarenal (45.5%), juxtarenal (36.4%), and suprarenal (18.1%) [13].

Open surgical repair of AAA is preferable, and EVAR is reserved for patients having hostile abdomen, anesthetic risk, and/or co-morbidities. All our participants received an open transabdominal repair of AAA [20]. We used PTFE for most patients as patency of graft and survival after open AAA repair with dacron vs. PTFE is found to be similar. But experimental studies have found PTFE to be less prone to thrombosis and infection and showed better adhesion and improved pliability as compared to Dacron grafts [21]. In our study, nine patients had infrarenal aortic clamping and eight patients had less than 45 minutes of clamping time. In three patients, clamping time exceeded 45 minutes, and all of them were smokers. Suprarenal clamping was done in two patients due to unfavorable neck anatomy. The average safer clamping time for AAA open repair surgeries is less than 45 minutes [22]. Prolonged clamping time in open repair surgeries may lead to cardiac or renal dysfunction or unclamping shock-hypotension. We encountered unclamping shock hypotension in one case of suprarenal AAA and was managed with adequate fluid resuscitation and administration of vasopressors, phenylephrine, and vasopressin in ICU. Smoking increases the risk of intraoperative complications like increased blood loss and may lead to an increase in clamping duration [8,19,23].

Postoperative complications in our patients included superficial skin infection in two and ileus in two patients. The rate of postoperative complications increases with age and due to smoking [24-25]. However, ileus remains the most common complication of abdominal surgery. It is usually temporary and resolves within 2-3 days [25]. Though not studied exclusively for young adults undergoing AAA repair, the etiology of ileus mainly results due to intraoperative tissue trauma, which may be independent of age.

The median length of hospital stay for patients in our study was 10 (7-18) days. The median LOHS was found to be more among patients with low to moderate physical activity and who had suprarenal and juxtarenal location of AAA in our study. These are in sync with the other studies, which also reported that older age, suprarenal and juxtarenal location, as well as the thoracoabdominal extent of aneurysm, are predictors of prolonged hospital stay [23]. The role of physical activity in faster recoveries is unclear, even though preoperative exercise therapy has shown beneficial effects on certain parameters [25]. In our study, three (27%) patients were kept in the ICU, and in two patients of infrarenal AAA, one had hypotension due to excessive blood loss and another patient was not extubated due to poor respiratory effort, one patient of symptomatic supra-renal AAA experienced unclamped shock hypotension and had an ICU stay in the postoperative period. Patients with open AAA repair, having comorbid conditions, or old age need an ICU stay as documented in another study [24].

During follow-up, no mortality was reported in our patients. This finding is in contrast to the one reported by Muluk et al. in which younger adults had reported higher 30-days mortality as compared to the elderly. However, due to the scarcity of case series on younger adults, there is insufficient evidence regarding mortality in the literature in this age group. However, preoperative aneurysmal rupture and renal insufficiency are associated with high 30-day mortality among the elderly, ranging from 65% to 85% with cause-specific mortality of 1.3% [20].

To the best of our knowledge, this is the first case series presenting a clinical presentation, risk factors, especially the effect of physical activity, and treatment outcomes following surgery among young adults in India. As we reported zero mortality following surgery among young adults, which is also in contrast to the usual outcomes of treating AAA among the elderly, this will give clinicians the motivation for operating on the patient as soon as possible.

The limitation of this study could be the absence of a control group having elderly patients of AAA so that we could compare the two groups for all patient characteristics and treatment outcomes. This limitation is a potential research area for future clinicians. Another limitation is the small sample size due to which we couldn’t use statistical tests for exploring the association of potential factors like smoking and level of physical activity and the presence of co-morbidities. As AAA among younger adults is a rare disease, we strongly recommend collaborative multicentric hospital-based case-control studies for achieving a sufficiently powered sample size.

## Conclusions

The presentation of an aneurysmal disease is usually symptomatic in young adults with abdominal pain as the main complaint rather than an aneurysmal rupture, which is a main clinical feature in the elderly. Our study suggested that smoking may play a role in increasing the median clamping time and length of stay in hospital. No mortality points to a better prognosis in young adults. Nevertheless, comprehensive, hospital-based, multi-center case studies may help clinicians explore these associations.
